# Anesthetic management using peripheral nerve block in patients with factor XI deficiency: a case report

**DOI:** 10.1186/s40981-016-0047-4

**Published:** 2016-08-08

**Authors:** Sachio Yoshikawa, Isao Kumano, Yasushi Satoh, Shinya Yufune

**Affiliations:** 1 Department of Anesthesiology, Japan Self Defense Forces Hospital Yokosuka, 1766-1 Tauraminatocho, Yokosuka, Kanagawa 237-0071 Japan; 2 Department of Surgery, Japan Self Defense Forces Hospital Yokosuka, 1766-1 Tauraminatocho, Yokosuka, Kanagawa 237-0071 Japan; 3 Department of Pharmacology and Department of Anesthesiology, National Defense Medical College, 3-2 Namiki, Tokorozawa, Saitama 359-8513 Japan

**Keywords:** Factor XI deficiency, Bleeding tendency, Anesthetic management

## Abstract

Factor XI deficiency is rare but may cause life-threatening bleeding during the perioperative period. The clinical manifestation of factor XI deficiency is characterized by bleeding tendency. This unpredictable bleeding tendency makes anesthetic management difficult. We report a case of a partial duodenectomy in a patient with factor XI deficiency. The patient was scheduled for duodenectomy because of a duodenal tumor. When checked for coagulation before surgery, the patient was found to have a remarkably prolonged activated partial thrombin time, and further investigation revealed factor XI deficiency. Fresh frozen plasma was transfused before surgery, and general anesthesia and peripheral nerve block were performed. In the present case of factor XI deficiency, supplementation with clotting factor and proper anesthetic management were important to prevent severe complications.

## Background

Factor XI is a plasma glycoprotein that participates in the intrinsic pathway of the blood coagulation cascade. Factor XI deficiency is a rare, autosomal recessive disorder present in 1:1,000,000 individuals [1]. In hemophilia A and B, the bleeding tendency is clearly related to factor level; however, a deficiency of factor XI results in unusual coagulopathy, with a poor correlation between the measured factor XI level and bleeding tendency [2]. The unclear presentation of factor XI deficiency results in difficulties in anesthetic management of patients with this disorder. In this report, we present a patient with factor XI deficiency who underwent duodenectomy for a duodenal tubulovillous adenoma.

## Case presentation

A 75-year-old woman (46.2 kg, 153.7 cm tall, and American Society for Anesthesiologists physical status II) was scheduled for a partial duodenectomy. She provided written informed consent for participation and publication. Her previous surgical history included ovariectomy under general anesthesia at the age of 38 years, for which the surgical and anesthetic procedures were uneventful. She also had a history of hypertension, which was treated with angiotensin II receptor blockers. She had no other past medical history. At presentation, she was not taking any herbal preparations or anticoagulants. She had no family history of bleeding tendency.

Four months prior to her surgery, she developed epigastric pain and an endoscopic examination revealed a duodenum tumor; she was scheduled for a partial duodenectomy. Laboratory findings on admission were as follows: normal electrocardiogram and chest X-ray, hemoglobin 13.4 g/dL, platelet count 21.5 × 10^4^ μL, and the coagulation tests showed prolonged activated partial thromboplastin time (APTT 89.7 s); however, the prothrombin time and the international normalized ratio of prothrombin time were normal (PT 10.3 s, PT-INR 1.01). Hemophilia A and B are usually considered first, especially in males, whenever a prolonged APTT is noted; however, our female patient had no family history of bleeding. Further investigation revealed severely deficient levels of factor XI, and factor XI deficiency was diagnosed. The levels of activity of her factors VIII, IX, XI, and XII are shown in Table 1.

**Table 1:** Coagulation factor levels

Factor	Activity level (%)	Reference range (%)
VIII	109	60–150
IX	84	70–130
XI	<3	75–145
XII	55	50–150

It is obvious that patients with severe clotting factor deficiencies are at risk of bleeding from the surgical site, and anesthetic management is one of the most important concerns for the attending anesthesiologist. To avoid massive bleeding during the surgical procedure, 4 units of fresh frozen plasma (FFP) was transfused (in Japan, the estimated volume per unit of FFP is 120 mL/unit) 1 day prior to the surgery. We performed another coagulation test immediately before entering the operating room, and her APTT level improved to 36.2 s.

In our facility, epidural anesthesia is the first choice of analgesia for open upper abdominal surgery. However, because the patient had a potential risk of epidural hemorrhage, we avoided epidural anesthesia and selected ultrasound-guided subcostal transversus abdominis plane (TAP) block with rectus sheath block. General anesthesia was induced by target-controlled infusion of propofol at a target plasma concentration of 3 μg/mL. Fifty milligrams of rocuronium was administered after loss of response to verbal commands. Remifentanil was initiated at a rate of 0.3 μg/kg/min, and propofol was adjusted to maintain a bispectral index of 40–60. Subcostal TAP block with rectus sheath block was performed in the supine position. After skin preparation with 0.5 % chlorhexidine, the ultrasound probe was placed parallel to the subcostal margin near the xiphoid process, the needle was advanced to the TAP, and 20 mL of 0.25 % levobupivacaine was administered to the TAP. After subcostal TAP block, the rectus muscle was imaged with the ultrasound probe on the level of the umbilicus and the needle was advanced to the posterior rectus sheath, and 10 mL of levobupivacaine was administered.

The surgical procedure itself was uneventful, and recovery was satisfactory. The operation duration was 289 min, and the anesthesia duration was 406 min. The blood loss was 222 mL, and the urinary output was 1250 mL. The patient received 3600 mL of fluid administration. The patient reported no pain at the surgical site after extubation. Postoperative supplemental analgesics were administered. The patients received intravenous flurbiprofen axetil and acetaminophen on the first postoperative day. After started oral intake, loxoprofen sodium was administered and reported no pain.

**Fig. 1:**
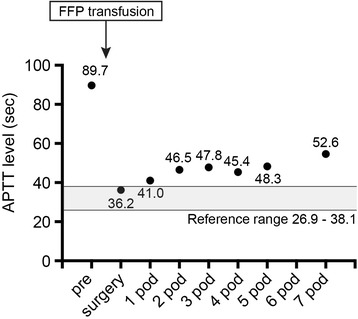
Perioperative activated partial thromboplastin time (APTT) levels. *FFP* fresh frozen plasma, *POD* postoperative day

After the surgery, thin, watery-pink exudate was seen from surgical drain, but on the seventh postoperative day, the color of the fluid changed to have a more slightly reddish consistency. Although amount of exudate from the surgical drain did not increase, the drain was removed on the same day. The levels of exudate from the surgical drain after surgery are shown in Table 2. Coagulation tests were performed every day after the surgery. The PT and PT-INR levels were normal throughout the perioperative period. The perioperative APTT levels are shown in Fig. 1.

**Table 2:** The levels of exudate from the surgical drain

POD	0	1	2	3	4	5	6	7
Exudate (mL)	95	140	134	162	178	141	120	25

### Discussion

Factor XI deficiency, which is relatively common among Ashkenazi Jews, is associated with injury-related bleeding [3]. This is sometimes called “hemophilia C,” is distinguished from hemophilia A and B by the absence of hemorrhage inside joints and muscles, and has equal incidence in individuals of either gender. The level of the deficiency does not determine the bleeding risk [4], and bleeding tendency varies among individuals. Patients who have severe factor XI deficiency are at risk of massive hemorrhage; however, some do not have this tendency [5].

Patients with factor XI deficiency need specific management during surgery. Previously, adverse surgical outcomes of patients with factor XI deficiency have been reported, including cerebral hemorrhage [6] and spinal epidural hematoma [7]. Different surgical management strategies, each with varying degrees of risk, have been reported, including FFP, factor XI concentrates, and desmopressin [8].

To manage hemorrhage tendency during the perioperative period, anesthesiologists must determine an appropriate method and tailor it to each patient. We used 4 units of FFP prior to the surgical procedure. The estimated half-life of factor XI is 45 h, which is similar to standard FFP [9]; therefore, we judged FFP transfusion 1 day prior to surgery which would prevent intraoperative and acute phase massive hemorrhage. As shown in Fig. 1, APTT levels improved from 89.7 to 36.2 s after FFP transfusion. We investigated the transition level of APTT after surgery. The level of APTT was gradually prolonged to 52.6 s by postoperative day 7. After the surgery, the color of the exudate fluid from surgical drain had changed to slightly reddish, which may have been related to the change of APTT. Considering the estimated half-life of factor XI, we anticipated that APTT would rise to the same level as the preoperative value. Fortunately, APTT remained shorter than expected, and we did not need to use additional FFP.

The disadvantages of plasma which are the large volumes are required and the potential for allergic reactions and infections. The patient described in our case report had no cardiac disease, and only 4 units of FFP were used, so fluid overload was not an issue; however, FFPs should be transfused carefully, especially in patients who have cardiac failure. Although factor XI concentrates are an effective treatment, they are not currently available in Japan.

In addition to medication, safe and reliable anesthesia is required to manage perioperative hemorrhage. Epidural anesthesia is one of the most useful methods to alleviate surgical pain during the perioperative period. Although the incidence of neurologic dysfunction resulting from hemorrhagic complication associated with neuraxial blockade, including epidural anesthesia, is unknown [10], the risk of bleeding is increased, especially in the presence of coagulopathy. In recent years, peripheral nerve blocks using ultrasound have gained popularity. It serves as an effective alternative analgesia when an epidural is contraindicated or refused. Although guidelines for regional anesthesia in the patient receiving anticoagulants are available, regional anesthesia for patients with a clotting factor deficiency is dependent on the careful decision-making by the attending anesthesiologist. In this case, we opted to avoid the potential risk of a spinal hematoma associated with epidural anesthesia and decided to control perioperative pain using a peripheral nerve block. Subcostal TAP block provides analgesia in the upper abdominal area [11], and combined with a rectus sheath block that provides effective pain relief for the umbilical are, we were able to alleviate pain in our patient. Neurologic dysfunctions after epidural anesthesia are very rare but devastating. Appropriate management for patients with clotting factor deficiency should result in an uneventful recovery.

## Conclusions

In conclusion, although factor XI deficiency is rare, it is sometimes diagnosed by unsuspecting patients prior to major surgery. Massive hemorrhage occurs unpredictably during perioperative period regardless of the bleeding tendency before surgery. FFP transfusion prior to surgery may help patients’ coagulation, and proper anesthetic management, including peripheral nerve block, may contribute to improved prognosis.

## Abbreviations

APTT, activated partial thromboplastin time; FFP, fresh frozen plasma; POD, postoperative day; PT, prothrombin time; PT-INR, international normalized ratio of prothrombin time; TAP, transversus abdominis plane
